# Genome-Wide Mapping of 5mC and 5hmC Identified Differentially Modified Genomic Regions in Late-Onset Severe Preeclampsia: A Pilot Study

**DOI:** 10.1371/journal.pone.0134119

**Published:** 2015-07-27

**Authors:** Lisha Zhu, Ruitu Lv, Lingchun Kong, Haidong Cheng, Fei Lan, Xiaotian Li

**Affiliations:** 1 Obstetrics Department, Obstetrics and Gynecology Hospital of Fudan University, Shanghai, China, 200011; 2 Key Laboratory of Epigenetics of Shanghai Ministry of Education, School of Basic Medicine and Institutes of Biomedical Sciences, Shanghai Medical College of Fudan University, Shanghai 200032, China; 3 Key Laboratory of Birth Defect, Children’s Hospital of Fudan University, Shanghai 201102, China; VU University Medical Center, NETHERLANDS

## Abstract

Preeclampsia (PE) is a leading cause of perinatal morbidity and mortality. However, as a common form of PE, the etiology of late-onset PE is elusive. We analyzed 5-methylcytosine (5mC) and 5-hydroxymethylcytosine (5hmC) levels in the placentas of late-onset severe PE patients (n = 4) and normal controls (n = 4) using a (hydroxy)methylated DNA immunoprecipitation approach combined with deep sequencing ([h]MeDIP-seq), and the results were verified by (h)MeDIP-qPCR. The most significant differentially methylated regions (DMRs) were verified by MassARRAY EppiTYPER in an enlarged sample size (n = 20). Bioinformatics analysis identified 714 peaks of 5mC that were associated with 403 genes and 119 peaks of 5hmC that were associated with 61 genes, thus showing significant differences between the PE patients and the controls (>2-fold, *p*<0.05). Further, only one gene, *PTPRN2*, had both 5mC and 5hmC changes in patients. The ErbB signaling pathway was enriched in those 403 genes that had significantly different5mC level between the groups. This genome-wide mapping of 5mC and 5hmC in late-onset severe PE and normal controls demonstrates that both 5mC and 5hmC play epigenetic roles in the regulation of the disease, but work independently. We reveal the genome-wide mapping of DNA methylation and DNA hydroxymethylation in late-onset PE placentas for the first time, and the identified ErbB signaling pathway and the gene *PTPRN2* may be relevant to the epigenetic pathogenesis of late-onset PE.

## Introduction

Preeclampsia (PE), a serious complication of pregnancy, is characterized by hypertension and proteinuria after 20 weeks of gestation in previously normotensive women[1]. This condition remains one of the leading contributors to perinatal morbidity and mortality, especially in developing countries. Although the etiology of PE is heterogeneous, PE is discriminated into 2 different disease entities: late-onset PE and early-onset PE[2]. These two entities require separate study, because they have different pathophysiology. Late-onset PE is likely maternal PE, i.e, the result of a maternal-inflammatory response, whereas early-onset PE is predominantly due to placental insufficiency [3, 4].

Emerging evidence indicates that epigenetic alternations, especially abnormal DNA methylations, are well-recognized hallmarks of PE[5, 6]. The novel DNA based 5-hydroxymethylcytosine (5hmC) is formed from the oxidation of 5mC by ten-eleven translocation (TET) enzymes, as an intermediate in passive or active DNA demethylation[7, 8]. In mammals, the level of 5hmC can be detected in almost all tissues and cell types [9, 10]. As an epigenetic modification that is indirectly involved in the regulation of gene expression [11, 12], 5hmC plays a direct role in gene transcription [13]. Several studies [14–16] have found 5hmC alternations in the epigenetic regulation of various diseases, including hypertension [17]. Because early-onset PE has been reported to have a common phenomenon of gene-specific hypomethylation in promoters and a significantly higher genome-wide methylation [18, 19], studying the DNA methylation changes in these abnormal placentas may uncover some clues about the underlying etiology of PE. Little is known about its DNA methylation in late-onset PE, and no report have been published about its 5hmC distribution. Because the distribution of 5hmC in several human tissues does not correlate with global 5-methylcytosine (5mC) content [20], and because the traditional bisulfide-sequencing methods are unable to distinguish 5mC from 5hmC, new approaches are necessary to reveal the involvement of 5mC and 5hmC in PE.

In an attempt to explore the epigenetic alternations, especially 5hmC changes, in late-onset PE, we performed genomic-wide mapping of 5mC/5hmC in placentas of late-onset severe PE patients, compared with those from normal patients, using a (hydroxy)methylated DNA immunoprecipitation ([h]MeDIP) approach combined with deep sequencing ([h]MeDIP-seq).

## Materials and Methods

### Patient samples and clinical data

Freshly-frozen placental samples were obtained from Han Chinese women who were delivered by elective caesarean section. Ethical approval for the collection of human placentas was granted by the Human Ethics Committee of the Obstetrics and Gynecology Hospital affiliated with Fudan University, and informed consent was obtained from all patients participating in this study. PE and severe PE were diagnosed according to the 2013 ACOG guidelines [21]. The criteria for exclusion were multiple pregnancies, pre-existing hypertension, diabetes mellitus, cardiac insufficiency, renal disease, HIV infection and preterm. Placentas from 20 late-onset severe PE cases and 20 normal pregnancies were collected, and detailed clinical information is provided in S1 Table. For (h)MeDIP-seq analysis, four samples of pregnancies complicated by late-onset severe PE and four samples of normotensive pregnancies were recruited, the severely preeclamptic women were parity and gestation matched with controls. The related clinical details for the selected patients and uncomplicated pregnancies are outlined in Table 1. Samples 7, 14 and 15 were diagnosed as severe PE because of their high BP and sample 40 was diagnosed due to the symptom of headache and visual disturbance.

**Table 1:** Demographic characteristics of the late-onset severe preeclampsia (Case) group and normotensive pregnancies (Control) group used for (h)MeDIP-seq.

Characteristic	Case group (sample number)				Control group (sample number)				*p*
	7	14	15	40	9	10	27	28	
Maternal age(years)	29	26	33	23	28	25	26	26	0.554
Gravidity(n)	4	1	1	1	3	2	1	1	0.741
Parity(n)	1	0	0	0	1	0	0	0	1.000
Gestation at delivery (weeks)	38.3	37.6	37.7	39.4	37.9	39.0	38.4	39.3	0.469
Pregnancy BMI(kg/m^2^)	30.4	33.2	26.0	25.4	27.7	24.4	29.2	31.2	0.799
Systolic BP(mmHg)*	160	161	166	145	96	129	122	103	0.002
Diastolic BP(mmHg)*	100	110	100	94	63	87	72	71	0.003
Hemoglobin(g/L)	101	116	112	122	105	142	115	125	0.358
Platelets (x10^9^/L)	282	166	203	149	118	181	197	172	0.372
AST (U/L)	14	22	13	15	12	31	16	11	0.777
ALT(U/L)	10	17	22	12	17	21	14	19	0.447
LDH(U/L)	204	213	432	468	393	499	451	215	0.544
Scr (μmol/L)	53	38	52	56	34	47	45	47	0.191
BUN(mmol/L)	3.9	3.2	3.1	2.7	3.1	4.2	3.5	4.0	0.226
Proteinuria(g/24 h)*	2.91	0.95	0.36	1.44	0.05	0.04	0.05	0.06	0.020
Infant birthwight(g)	3580	3510	3265	3140	3490	4215	2590	3270	0.962
5-min Apgar score	9	9	9	9	10	9	9	9	0.317
1-min Apgar score	9	9	9	9	9	9	9	9	1.000
Umbilical artery S/D rate	1.64	2.03	1.95	2.13	2.16	2.29	2.38	2.05	0.070

* represents statistical significance.

ALT: alanine transaminase; AST: aspartate transaminase; LDH: lactic acid dehydrogenase; BUN: blood urea nitrogen; Scr: serum creatinine.

### DNA extraction and sodium bisulfite conversion

Genomic DNA was extracted from frozen placental tissues using the DNeasy Blood and Tissue kit (Qiagen, Valencia, CA) according to the manufacturer's protocol. Briefly, placental tissue was homogenized using a hand-held homogenizer, digested with Proteinase K (Qiagen) and RNase A (Qiagen, Valencia, CA) overnight at 56°C, precipitated and washed. The concentration and purity of DNA were measured using a NanoDrop 1000 Spectrophotometer (Thermo Scientific, MA, USA). DNA samples for quantitative DNA methylation analysis by MassARRAY EpiTYPER were bisulfite converted using the EZ DNA Methylation Kit (Zymo Research, CA, USA) according to the manufacturer’s instructions.

### MeDIP-seq and hMeDIP-seq

As described previously [22], genomic DNA from each placental tissue sample was sonicated to produce DNA fragments measuring less than 500bp. Illumina barcode adapters were ligated before MeDIP and hMeDIP. Approximately 4 mg of adaptor-ligated gDNA from the case and control samples were pooled together in one tube. IP buffer was used to denature and dilute the mixed DNA (10% was taken off as input at this step). The denatured DNA was incubated with 10 mg anti-5mC antibody (Active Motif, 39649) or 3 ml anti-5hmC antibody (Active Motif, 39770) at 4°C overnight. The antibody–DNA complexes were captured by protein A/G beads, and the hMeDIP and MeDIP products were purified and sequenced followed by standard Illumina protocols [23]. The image analysis and base calling were performed with the Illumina package CASAVA (v1.8.2). The raw sequence reads of hMeDIP, MeDIP and Input were separated into different files, according to the specific barcode sequences, reads were mapped to the human genome (NCBI Build UCSC hg19) using the Bowtie (v0.12.7) algorithm[24]. Significantly enriched regions (5hmC & 5mC peaks) were determined by MACS (v1.4) based on *p*<10^−5^ and FDR<0.01. The raw data file has been deposited to GEO (S2 Table).

### DMR and DHMR identification

DMR and DHMR identification are based on two independent methods. The first approach uses the R Bioconductor package DiffBind, which provides functions for processing ChIP-Seq data enriched for genomic loci where specific protein/DNA binding occurs, including peak-sets identified by ChIP-Seq peak callers and aligned sequence read datasets. The primary emphasis of this package is on identifying sites that are differentially bound between two sample groups. The other method is our own strategy, which is based on RPKM-normalized 5hmC and 5mC density, followed by Student’s t-test to compare case samples and control samples, after which we selected the differential 5hmC and 5mC regions with at least 2-fold density differences and *p*-values<0.05.

### MeDIP-qPCR and hMeDIP-qPCR

Input, MeDIP and hMeDIP products were used as templates for quantitative real-time PCR in an ABI PRISM7900HT system. The relative 5mC and 5hmC enrichment levels were calculated using the comparative CT method (2^dCt^ = 2^CtInput-Ct(h)MeDIP^), which determines the amount of target normalized to input. The primers used in MeDIP-qPCR and hMeDIP-qPCR are described in S3 Table.

### Gene ontology (GO) analysis and pathways analysis

The GO term and KEGG pathway analysis for the genes associated with DMRs and DHMRs were performed by the database for annotation, visualization and integrated discovery (DAVID) website[25].

### Quantitative MassARRAY analysis of gene methylation status

DNA methylation at selected gene DMRs was quantified with MassARRAY EpiTYPER assays (Sequenom) in an enlarged sample size (n = 20/group). The amplicons used in this study were designed using Methprimer (http://www.epidesigner.com) (S4 and S5 Tables). Each reverse primer was added an additional T7 promoter tag for in vivo transcription and a 10-mer tag was incorporated to the forward primer to adjust for the melting temperature differences. Inapplocable readings and their corresponding sites were excluded from the raw data. The average methylation ratios of the case and control groups were calculated as the mean value of the CpGs methylation rate and expressed as a relative amount of methylation.

### Statistical analysis

All statistical analyses were performed using the SPSS 17.0 statistical software. Either Student’s t test or the Mann-Whitney test was performed to evaluate the significance of any differences between the case and control samples. A two-tailed *p*<0.05 was considered to indicate statistically significant differences.

## Results

The two groups were comparable in terms of maternal age, gestational age at delivery, laboratory findings and the outcomes of the infants. As expected, the systolic BP, diastolic BP and proteinuria of late-onset severe PE patients were significantly higher than those of the controls (*p*<0.05).Other factors were not significantly different between two groups.

### Global DNA (hydroxy)methylation changes in severe PE tissues

**Fig 1:**
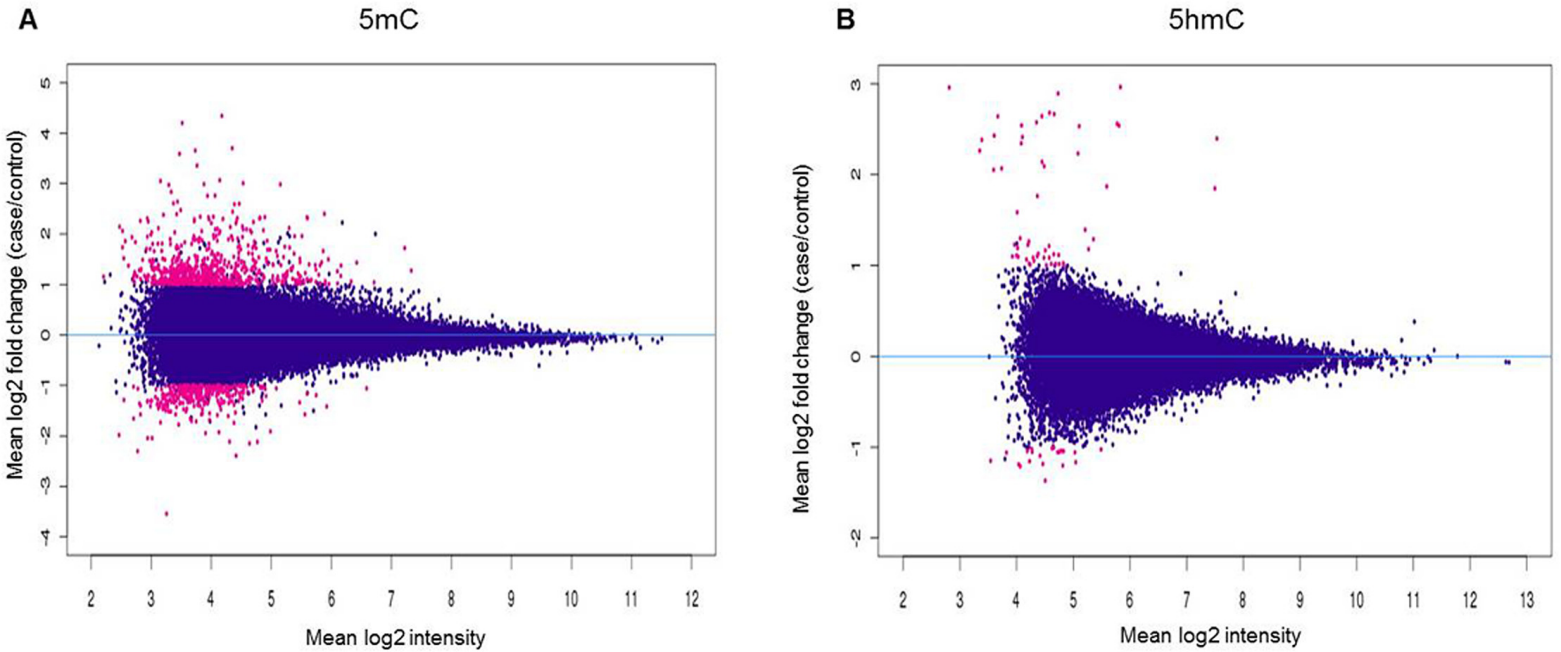
MA plot of Case-Control contrast of (A) 5mC peaks and (B) 5hmC peaks normalized with tag density. The X axis indicates the normalized mean and the Y axis indicates the log2-fold change. Red is used to indicate significantly differently expressed observations (at least 2-fold density changes and *p*-value<0.05). The blue dots show no differential expression between the two groups.

**Fig 2:**
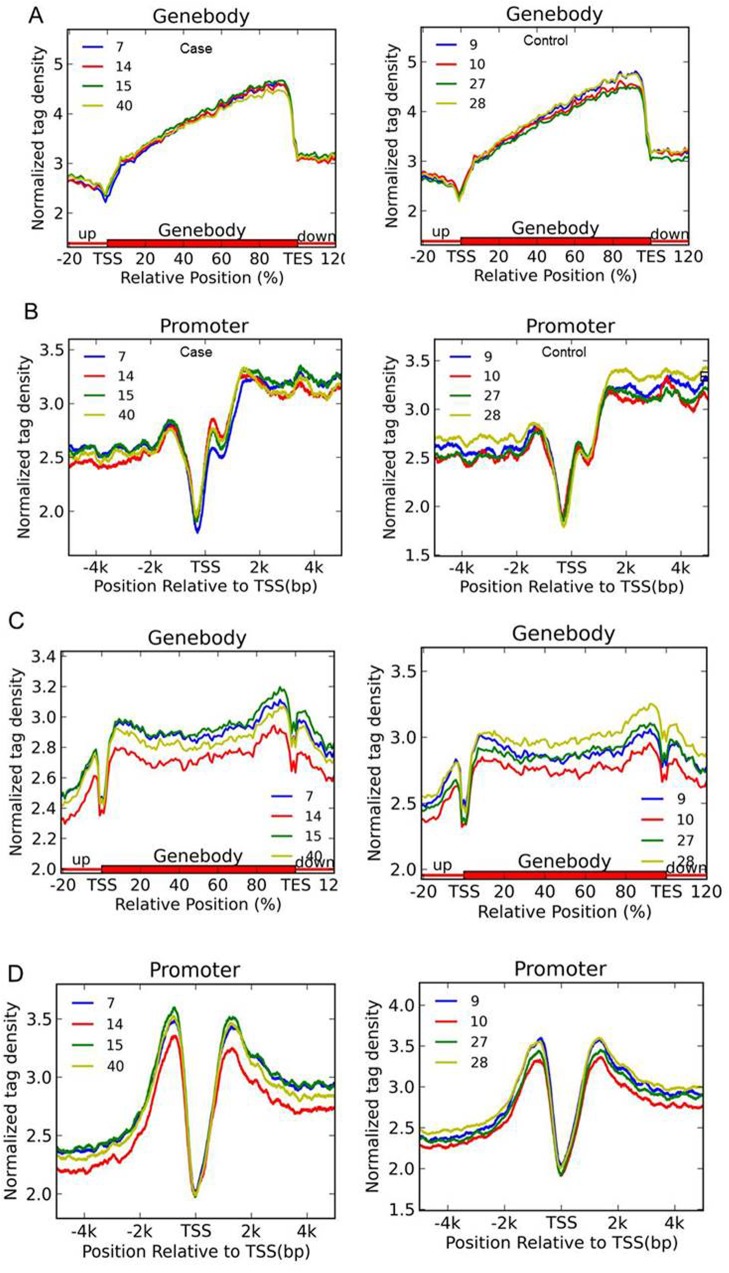
Genome-wide mapping of 5mC and 5hmC in placentas of late-onset severe PE and normal pregnant women. (A and C) Normalized DMR (A) and DHMR(C) tag density distribution across the gene body. Each gene body was normalized to 0%-100%. Normalized Tag density is plotted from 20% of upstream of TSSs to 20% downstream of TSSs(Transcription Start Sites). (B and D) Normalized DMR (B) and DHMR (D) tag density distribution at gene promoters. -5 kb to +5 kb relative to TSSs is shown.

We isolated total genomic DNA from all 8 samples, and employed a barcoded (hydroxy)methylated DNA immunoprecipitation ([h]MeDIP) approach combined with deep sequencing ([h]MeDIP-seq) to map genome-wide 5mC and 5hmC profiles for all 8 samples. Approximately 7.73 Gb and 9.91 Gb of sequencing data of 5mC and 5hmC were collected, respectively. Using the MACS software (*p*<10^−5^, FDR<0.01), we identified the 5mC and 5hmC peaks in each samples (Table 2). To compare the individuals in the case and control groups, 5mC and 5hmC tag densities were normalized by the total sequencing reads. Fig 1 shows an MA plot for a genome-wide comparison of 5mC/5hmC levels between the case and control groups. Most of the scatter plots are roughly symmetrical about the 0 axis, which means that 5mC/5hmC sites were similar between the groups. Thus, no significant genome-wide differences in the 5mC /5hmC densities between the case and control groups were found (Fig 1A and 1B). Next, we mapped the (h)MeDIP-seq signals of 5mC and 5hmC peaks according to their genomic location. Again, we did not observe any place at the gene body or promoter region with opposite 5mC/5hmC levels in the two groups, and the variation trend of the 5mC/5hmC levels had no significant differences between the groups (Fig 2A–2D).

**Table 2:** Pooled peak statistics for the hMeDIP and MeDIP data among 4 case samples (7,14,15,40) and 4 control samples(9, 10, 27, 28).

	Sample 7	Sample 14	Sample 15	Sample 40	Sample 9	Sample 10	Sample 27	Sample 28
5hmC peaks	47139	39964	49306	29241	31974	30388	33854	34453
5mC peaks	80441	79978	87865	58536	68895	63847	72472	66477

Macs1.4, FDR%< = 5

**Fig 3:**
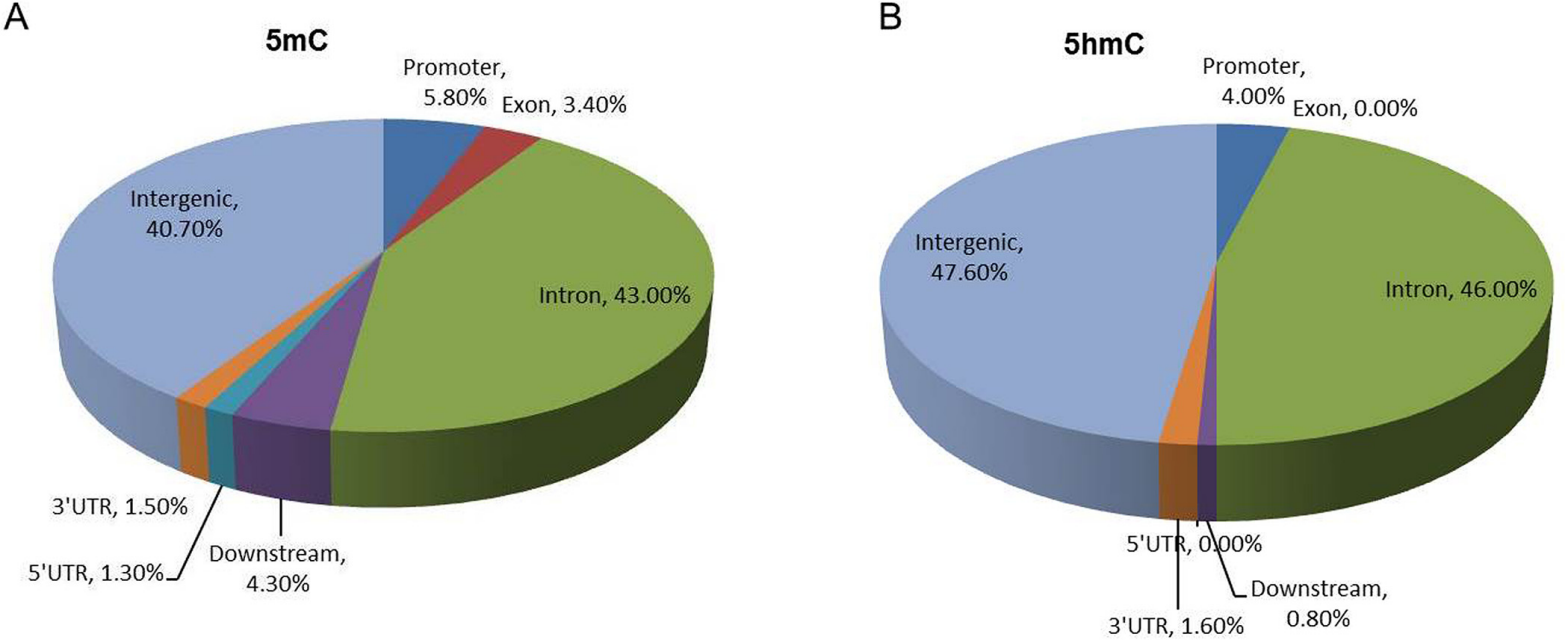
Genomic distributions of DMR (A) and DHMR (B) between case and control groups. The genomic features (extrons, introns, and intergenic regions) were defined based on RefSeq gene (hg19) annotations. The promoter was defined as -2 kb to +2 kb relative to TSS. UTR: Untranslated Regions.

**Fig 4:**
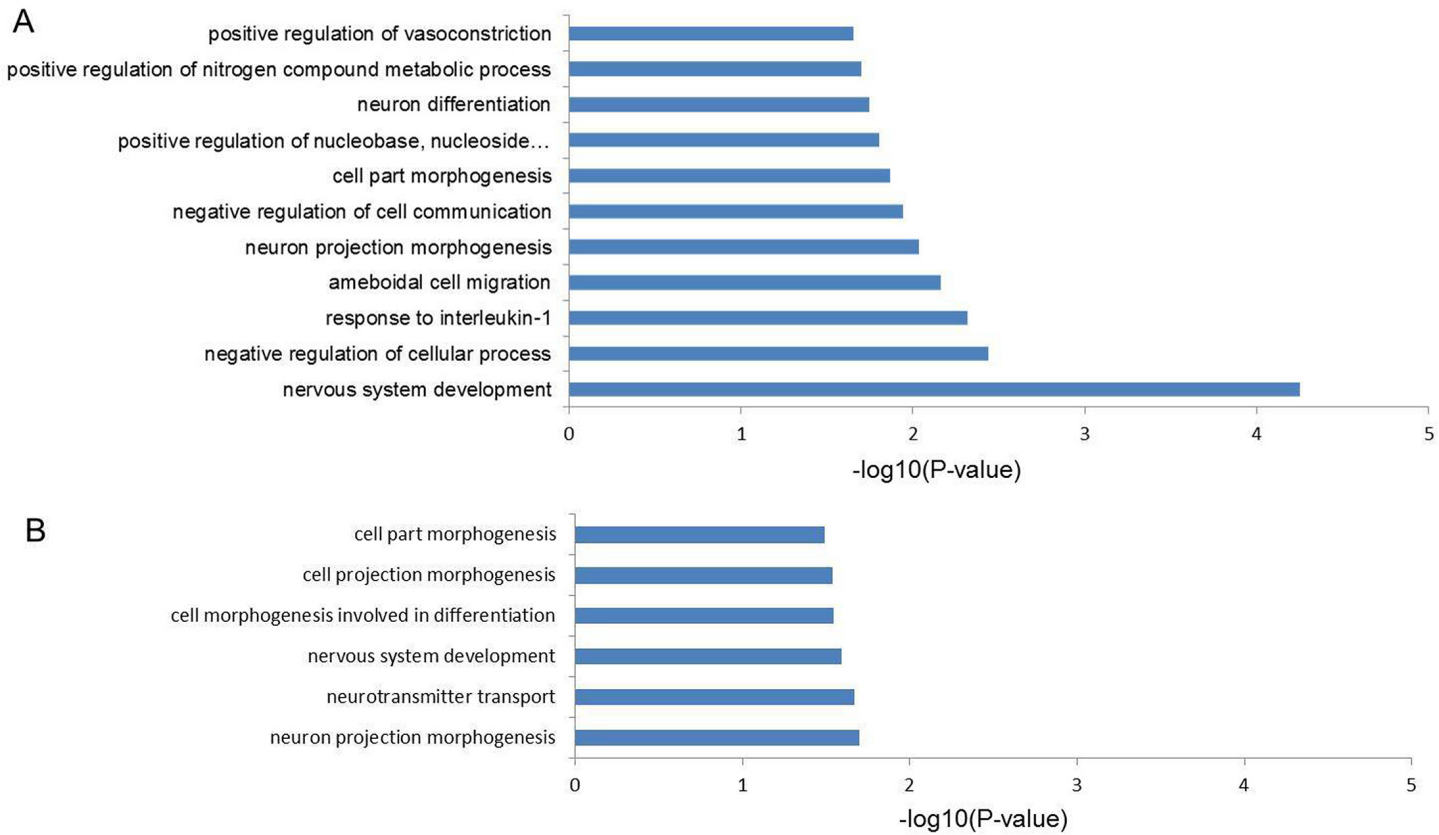
Gene ontology groups displaying the significant GO-terms of DMRs and DHMRs (p<0.05). (A)The significant GO-terms of DMRs between the groups. (B)The significant GO-terms of DHMRs between the groups.

Because we did not find global differences between the case and control groups, we carried out further bioinformatics analyses to identify locus-specific DMRs between the case and control samples. A total of 714 differential 5mC peaks (DMRs) were found showing significant difference between the two groups (>2-fold, *p*<0.05), 487 (68.2%) of them had higher 5mC levels in the late-onset preeclamptic placentas, and the genomic distribution of all 714 DMRs is shown in Fig 3A. These DMRs associated with 403 Refseq genes, of which 89 genes contained differential promoter 5mC (-2k to +2k of TSSs) and 75 showed higher 5mC levels at the promoter in the case group, 56 genes showed differential 5mC at both the promoter and the gene body between two groups (S6 Table). Gene ontology (GO) analysis of the 403 genes showed that the most significant GO category is nervous system development (*p* = 5.57×10^−5^), the other significant GO categories included negative regulation of cellular processes (*p* = 3.3×10^−3^) and response to interleukin-1 (*p* = 4.77×10^−3^) (Fig 4A). KEGG pathway enrichment analysis for 403 genes revealed that two pathways were enriched, including the ErbB signaling pathway (*p* = 3.83×10^−3^) and purine metabolism (*p* = 2.32×10^−2^).

The same analyses were carried out to search for differential 5hmC peaks (DHMRs) between case and control groups. A total of 119 DHMRs were identified, 64 (53.8%) of which had a higher level of 5hmC in the late-onset severe PE group. The genomic distribution of all 119 DHMRs is shown in Fig 3B. These 119 DHMRs were associated with 61 Refseq genes, of which 58 and 8 genes contained DHMRs in gene bodies and promoters, respectively, and 6 genes had higher 5hmC levels in promoter regions. These 8 genes were *AADACL3* (arylacetamide deacetylase-like 3), *CCDC149* (coiled-coil domain containing 149), *SLC35B4* (solute carrier family 35,member B4), *OTUD5* (OUT deubiquitinase 5), *RAB41* (member RAS oncogene family), *RAB5B* (member RAS oncogene family), *SERPINB7* (serpin peptidase inhibitor, clade B, member 7) and *KCNJ4* (potassium channel, inwardly rectifying subfamily J, member4). GO analysis identified an association with cellular component morphogenesis (*p* = 2.02×10^−2^), cell morphogenesis (*p* = 2.15×10^−2^) and regulation of neurotransmitter transport (*p* = 2.58×10^−2^) (Fig 4B). KEGG analysis did not identify any pathway that showed enrichment.

### Verification of DMRs and DHMRs between the case and control groups

**Fig 5:**
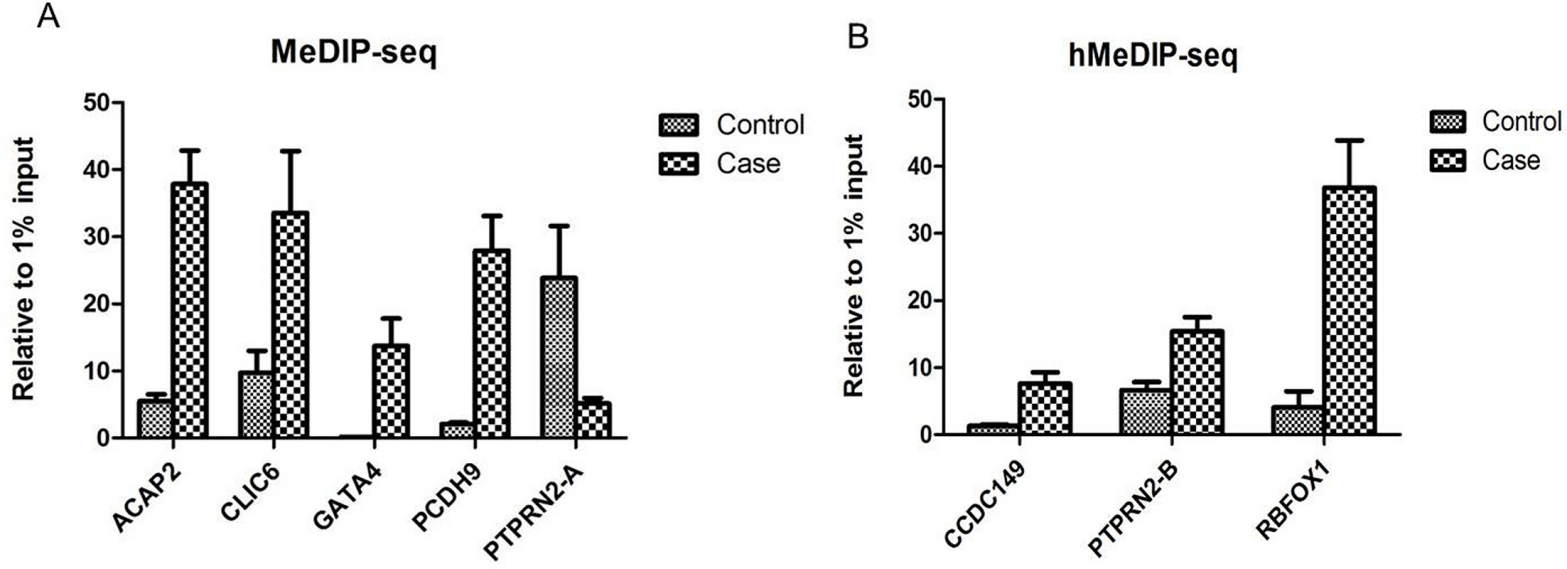
MeDIP-qPCR (A) and hMeDIP-qPCR (B) validations (mean values±SEM, n = 4 per group, *p*<0.05) of representative DMRs and DHMRs. (A) MeDIP-qPCR validation of *ACAP2*, *CLIC6*, *GATA4*, *PCDH9* and *PTPRN2*-A. (B)hMeDIP-qPCR validation of *CCDC149*, *PTPRN2*-B and *RBFOX1*. *ACAP2*:*p* = 0.0032; *CLIC6*: *p* = 0.0235; *GATA4*: *p* = 0.0429;*PCDH9*: *p* = 0.0081; *PTPRN2-A*: *p* = 0.0216; *CCDC149*: *p* = 0.0068; *PTPRN2-B*: *p* = 0.0018; *RBFOX1*: *p* = 0.0250.

The DMRs /DHMRs that had the top methylation/hydroxymethylation differences between the two groups and related to Refseq genes were selected for further verification by conventional (h)MeDIP-qPCR assays (S7 Table), and the densities of the 5mC/5hmC peaks in each group were normalized with 1% input before comparison. As shown in Fig 5, the 5mC peaks located in the *ACAP2*, *CLIC6*, *GATA4* and *PCDH9* genes exhibited gain-of-5mC in the case group compared with the control group, and the 5mC peaks in the *PTPRN2* gene (locus A) showed a decrease in the case group (*p*<0.05). The 5hmC peaks in *CCDC149*, *PTPRN2* (locus B is a different location from locus A) and *RBFOX1* genes were increased in the case group (*p*<0.05). The (hydroxy)methylation patterns of candidate DMRs/DHMRs, as verified by (h)MeDIP-qPCR, were consistent with the pattern identified by (h)MeDIP-seq, which suggests that our (h)MeDIP-seq results are reliable.

**Fig 6:**
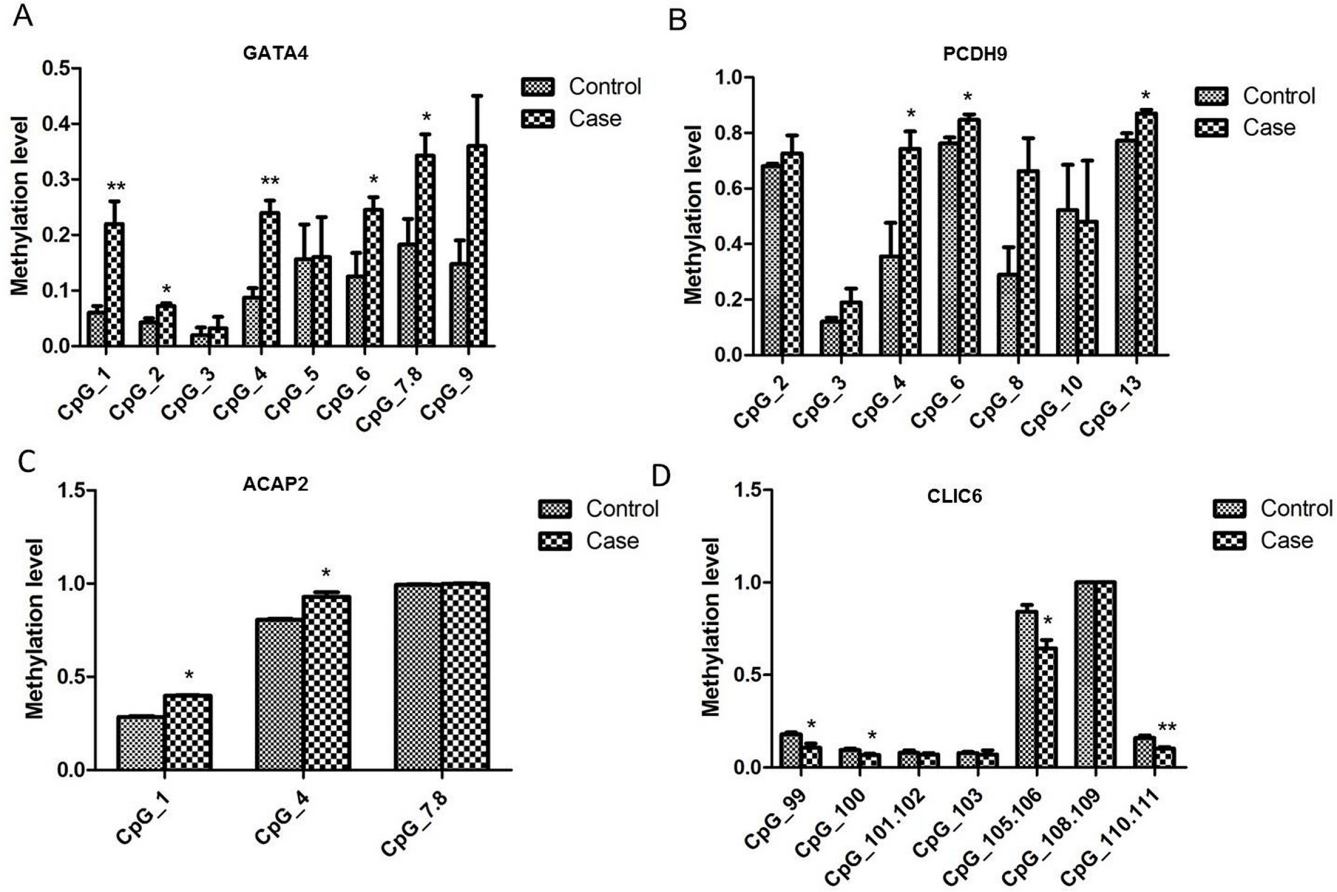
Validation of the methylation status of candidate DMRs between the late-onset severe PE group and the normal group by MassARRAY EpiTYPER. (A) The methylation level of the CpG sites within *GATA4* amplicon. (B) The CpG methylation level sites within the *PCDH9* amplicon. (C) The methylation level of the CpG sites within *ACAP2* amplicon. (D) The CpG methylation sites within *CLIC6* amplicon. Data are shown as the means±SEM, n = 20 per group, *p<0.05, **p<0.01. *GATA4* amplicon: CpG_1, *p* = 0.0093; CpG_2, *p* = 0.0150; CpG_3, *p* = 0.5993; CpG_4, *p* = 0.0015; CpG_5, *p* = 0.9738; CpG_6, *p* = 0.0481; CpG_7.8, *p* = 0.0377; CpG_9, *p* = 0.0661.*PCDH9* amplicon: CpG_2, *p* = 0.6177; CpG_3, *p* = 0.1831; CpG_4, *p* = 0.0294; CpG_6, *p* = 0.0265; CpG_8, *p* = 0.0529; CpG_10, *p* = 0.8818; CpG_13, *p* = 0.0158. *ACAP2* amplicon: CpG_1, *p* = 0.0316; CpG_4, *p* = 0.0349; CpG_7.8, *p* = 0.3524. *CLIC6* amplicon: CpG_99, *p* = 0.0446; CpG_100, *p* = 0.0424; CpG_101.102, *p* = 0.5504; CpG_103, *p* = 0.7697; CpG_105.106, *p* = 0.0310; CpG_108.109, *p* = 1.0000; CpG_110.111, *p* = 0.0084.

The 5mC status of *ACAP2* (chr3:195033505–195034277), *CLIC6* (chr21:36042019–36043071), *GATA4* (chr8:11561772–11563173), *PCDH9* (chr13:66919517–66920554) and *PTPRN2* (chr7:158129058–158130078) was further analyzed using the Sequenom MassARRAY approach in an enlarged sample size (n = 20/group). Four CpG sites among all 8 CpG sites within the *GATA4* amplicon and 3 CpG sites among all of the 7 CpG sites within the *PCDH9* amplicon showed significantly higher methylation levels in the case group (Fig 6A and 6B), whereas the *ACAP2* and *CLIC6* amplicons showed mildly increased methylation levels in the case samples (Fig 6C and 6D).

**Fig 7:**
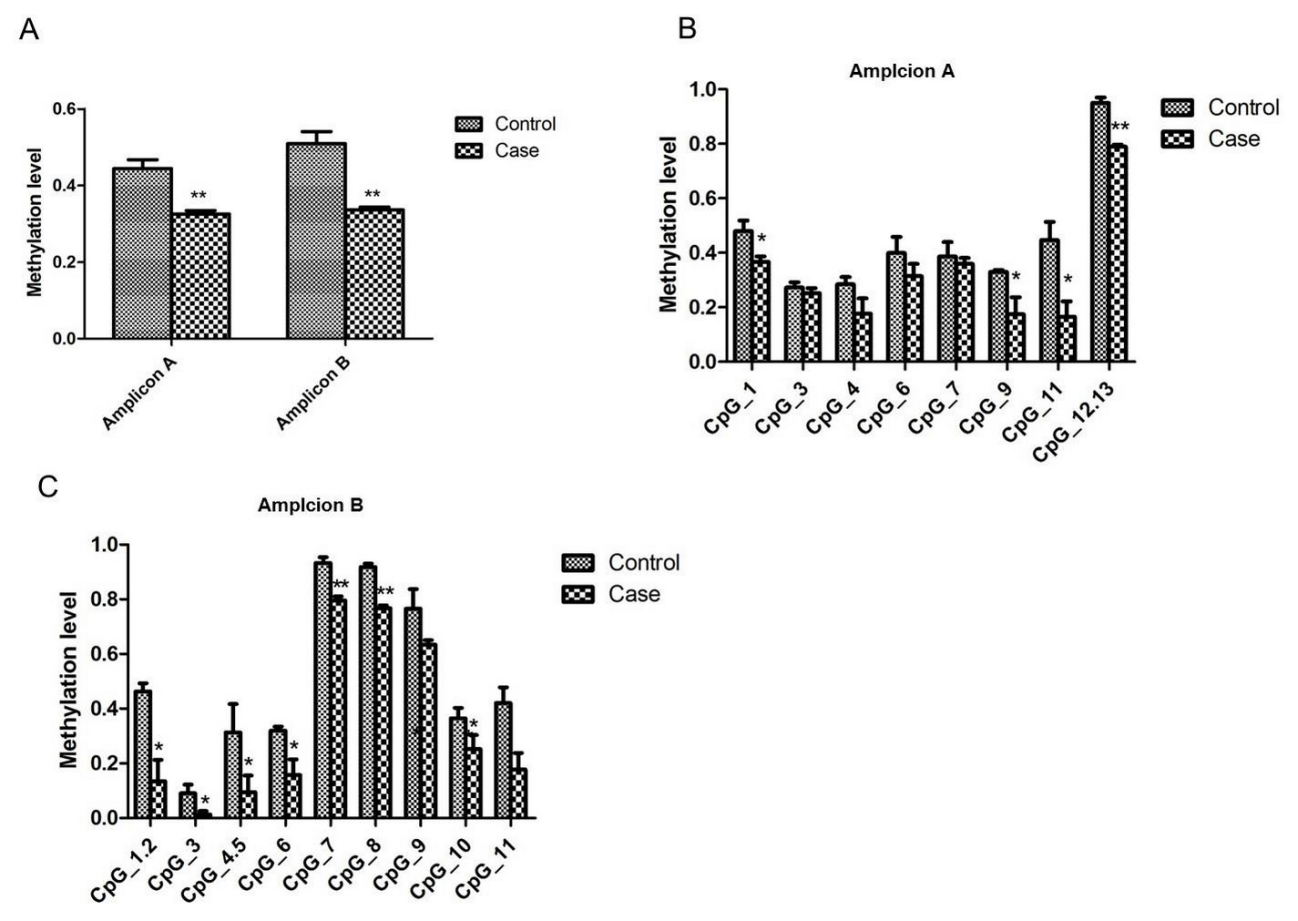
Validation of the methylation status of *PTPRN2* in the two groups by MassARRAY EpiTYPER. (A)The overall methylation levels are displayed within amplicon A and amplicon B. (B,C) The average methylation of the CpG units of amplicon A and amplicon B are presented for late-onset severe PE and normal patients. Data are presented as means±SEM, n = 20 per group, *p<0.05,**p<0.01.A: Amplicon A, *p* = 0.0033;Amplicon B, *p* = 0.0017.B: Amplicon A: CpG_1, *p* = 0.0420; CpG_3, *p* = 0.4820; CpG_4, *p* = 0.1296; CpG_6, *p* = 0.2957; CpG_7, *p* = 0.6410; CpG_9, *p* = 0.0475, CpG_11, *p* = 0.0177; CpG_12.13, *p* = 0.0003. C: Amplicon B: CpG_1.2, *p* = 0.0134, CpG_3, *p* = 0.0210, CpG_4.5, *p* = 0.0185; CpG_6, *p* = 0.0163; CpG_7, *p* = 0.0019; CpG_8, *p* = 0.0001; CpG_9, *p* = 0.1268; CpG_10, *p* = 0.0192; CpG_11, *p* = 0.0583.

Due to the features of the native sequence, the Methprimer program could not design proper pair for the *PTPRN2* DMR region, thus, we turned to the adjacent promoter region of *PTPRN2* (Amplicon A: chr7: 158381655–158382092; Amplicon B: chr7: 158381244-158381511- these had not been identified as differential regions between the groups by MeDIP-seq). For both of these regions, the average methylation level was significantly lower in the case group than in the control group (Fig 7A). Thirteen of the CpG sites fell within Amplicon A, and 4 of these sites were observed to have significantly lower 5mC levels in the case group than in the control group (Fig 7B). For Amplicon B, which contains 11 CpG sites, 7 of the sites showed significantly lower 5mC levels in the case group than in the control group (Fig 7C).

## Discussion

In this study, we show widespread alternations in DNA methylation and hydroxymethylation in placental tissues from late-onset severe PE and normal pregnancies, whereas the average 5mC and 5hmC levels exhibited no significant differences between the groups (Figs 1–3). The strengths of this study include the highly sensitive method and high-throughput sequencing used, which enabled the non-biased mapping of aberrant (hydroxy)methylation sites between the groups, and distinguished the alternation of 5mC from that of 5hmC. This is the first report on the genome-wide profiling of 5mC and 5hmC in late-onset severe PE placentas. The gene *PTPRN2* contains both 5mC and 5hmC alternations in the PE patients (Fig 5), and the enriched ErbB signaling pathway associated with the genes having differential methylation changes implies that epigenetic changes in the placenta can reveal the pathophysiology of PE.

Several studies have implicated alternations of 5hmC in cancer and some neurodevelopment diseases [26, 27]. We did not find changes in the overall levels of 5mC in late-onset severe PE, but 119 DHMRs were identified, 61 of the DHMRs were potentially related to gene expression, and 8 of them were found in gene promoter regions. Among these 8 genes, *SLC35B4* was a regulator of obestiy and glucose homeostasis[28, 29], which are the high risk factors for late-onset PE[30]. *OTUD5* reportedly regulates the activation of p53 in response to genotoxic stress[31], and the p53-pathway exaggerates apoptosis and autophagy in PE[32, 33]. Maternal *SERPINB7* was found to be a serum biomarker of early spontaneous preterm birth[34], and PE was associated with the fetal risk of death by preterm birth[35]. The discovery of these genes demonstrates that changes in 5hmC are related to the development of late-onset PE. In addition, no regions had both 5mC and 5hmC changes in the PE patients, thus revealing that 5mC and 5hmC may play independent roles in the development of late-onset severe PE.

Yuen et al. found no differentially methylated CpG loci in the placentas of late-onset PE compared with controls by Illumina microarray[36], but DNA hypermethylation or hypomethylation was found in many gene promoter regions of late-onset severe PE placentas. Moreover, the hypermethylation regions occurred more frequently than did hypomethylation, which means those relative genes may not express in late-onset severe PE. This finding is unlike the 5mC distribution in early-onset PE, which supports the idea that the etiology of early-onset PE is different from that of late-onset PE [3, 4]. Some of the genes that were found to be related to DMRs in our study have been previously reported in PE placentas, such as *ACOX2[37], ACVR2A*[38], *CD38*[39, 40], *CXCL12*[41, 42], *FLNB*[43], *GRB2*[18], *HSD17B1*[44], *LRP1*,[45], *IRAK3*[46], *PARD3*[47, 48], and *UTS2R*[49]. However, more than three hundred new genes were first found to have methylation changes in PE. Interestingly, a new gene *PTPRN2* was found to have decreased 5mC (>4-fold) and increased 5hmC (>2-fold) (Fig 5) in late-onset severe PE. The *PTPRN2* gene encodes a protein with phosphatase activity that functions in the TypeⅠdiabetes mellitus pathway. This gene also exhibits differential methylation in chronic kidney disease (CKD) which may relate to CKD complicated with hypertension and diabetes mellitus [50, 51]. So the gene *PTPRN2* we found here may have some relationship with the late-onset PE, but the specific mechanism it had needed further study. Regarding the pathway categories of the genes exhibiting methylation differences between the two groups, many of them were found to play roles in the ErbB signaling pathway, and inhibiting the ErbB pathway could lead to hypertension or poor trophoblast differentiation [52, 53]. Because hypoxia and the hypoperfusion caused by poor trophoblast differentiation are the recognized as components in the pathogenesis of PE, the ErbB signaling pathway may be a crucial path leading to the occurrence of late-onset PE.

One of the potential limitations of our study is the sample size, which may not be sufficiently large. This factor is mainly due to the high cost of (h)MeDIP-seq, which makes its use prohibitive on a large scale. The other limitation is that highly methylated repetitive DNA among captured fragments may influence the sensitivity of (h)MeDIP-seq, so the DMRs or DHMRs that we found may be false positives. To resolve this problem, we used (h)MeDIP-qPCR to validate the regions that had top 5mC/5hmC changes, and used MassARRY EpiTYPER to confirm the DMRs in an enlarged sample size.

In conclusion, our study, for the first time, profiled the genome-wide distributions of 5hmC and 5mC between placental tissue from late-onset severe PE patients and normal pregnancies. The broad 5hmC and 5mC changes may play independent roles in the development of late-onset severe PE. The discovered *PTPRN2* and the enriched ErbB signaling pathway revealed a possible route for the pathophysiology of late-onset PE. This pilot study provides a basis for further research with a larger sample size and clarifies the exact function that epigenetic changes play in the pathogenesis of late-onset PE.

## Supporting Information

S1 Table Demographic characteristics of the late-onset severe PE group (Case) and normotensive pregnancies group (Control). (TIF)

S2 Table The accession number for the results of (h)MeDIP-seq. (TIF)

S3 Table Primer sequences for (h)MeDIP-qPCR. (TIF)

S4 Table Methprimer sequences for MassARRAY analysis. (TIF)

S5 Table Methprimer sequences of *PTPRN2* for MassARRY analysis. (TIF)

S6 Table The genes which showed differential 5mC at both the promoter and the gene body between two groups. (TIF)

S7 Table The DMRs /DHMRs that had the top methylation/hydroxymethylation differences between the two groups and related to Refseq genes. (XLSX)
