# Supplementary figures and images for: Genome-Wide Mapping of 5mC and 5hmC Identified Differentially Modified Genomic Regions in Late-Onset Severe Preeclampsia: A Pilot Study
Source: PLoS One. 2015 Jul 27;10(7):e0134119. doi: 10.1371/journal.pone.0134119 (PMC4516306; doi:10.1371/journal.pone.0134119)

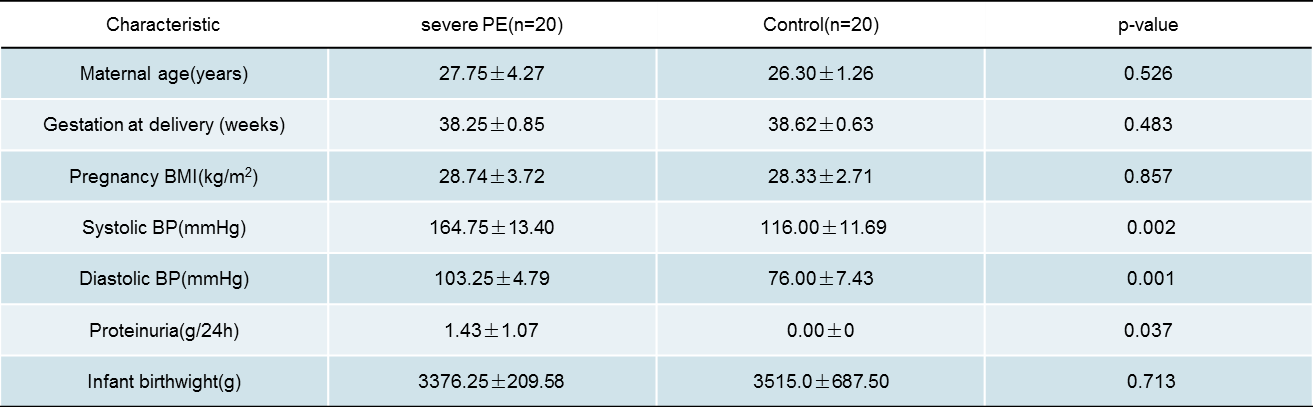

Supplement: S1 Table — (TIF) [file pone.0134119.s001.tif]

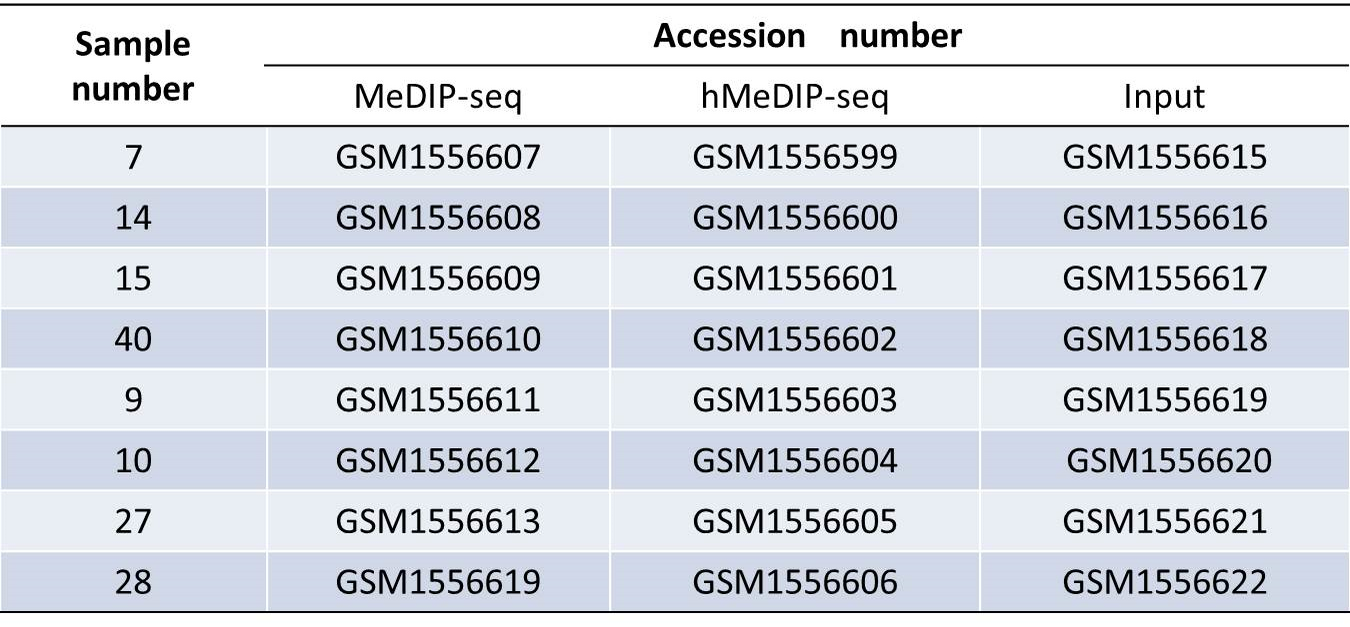

Supplement: S2 Table — (TIF) [file pone.0134119.s002.tif]

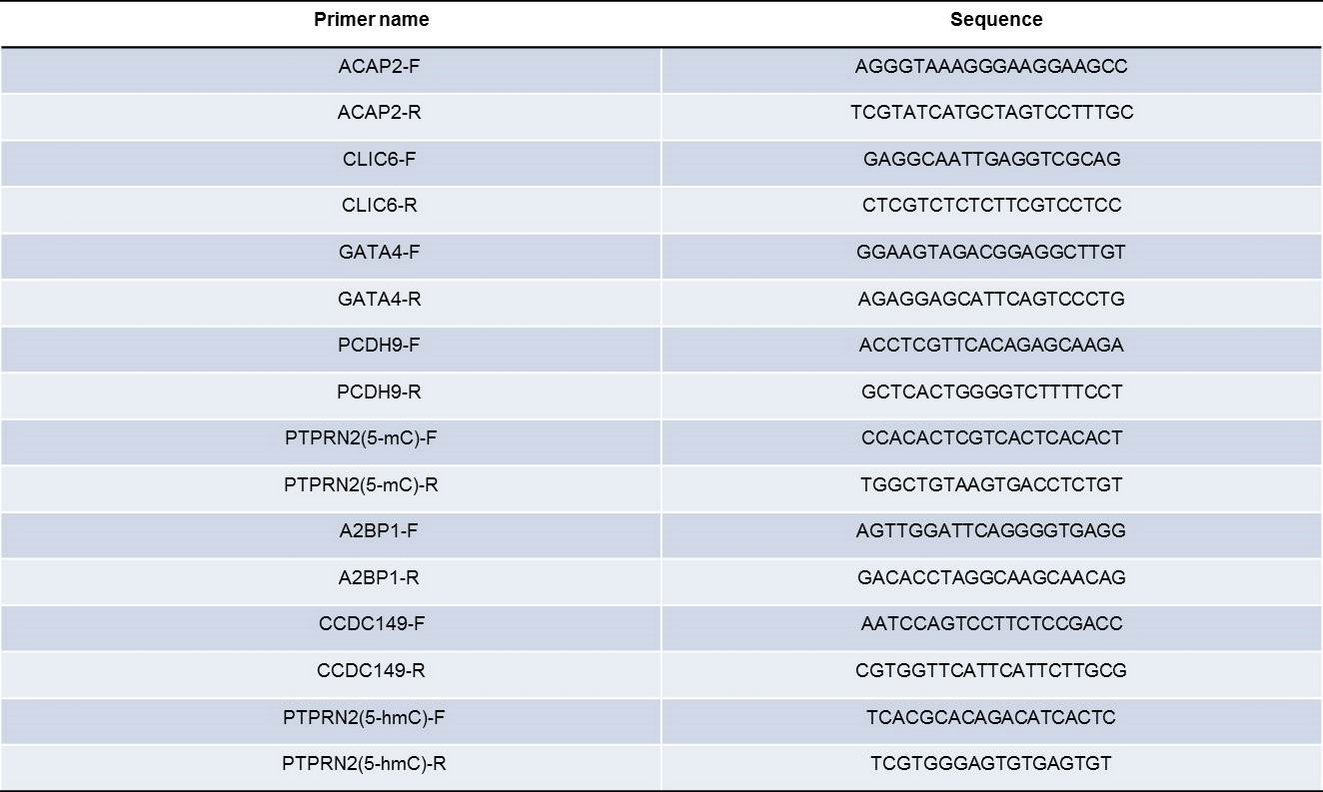

Supplement: S3 Table — (TIF) [file pone.0134119.s003.tif]

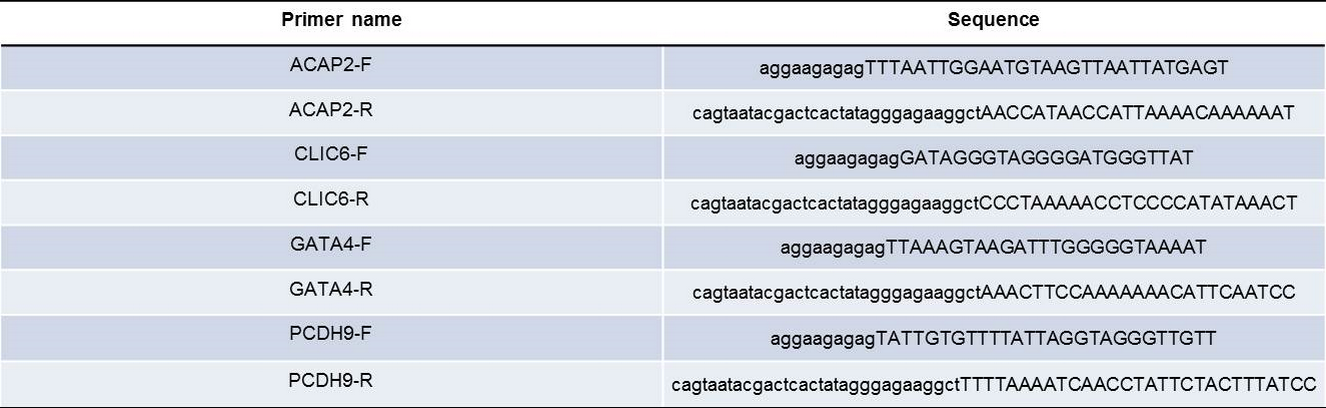

Supplement: S4 Table — (TIF) [file pone.0134119.s004.tif]

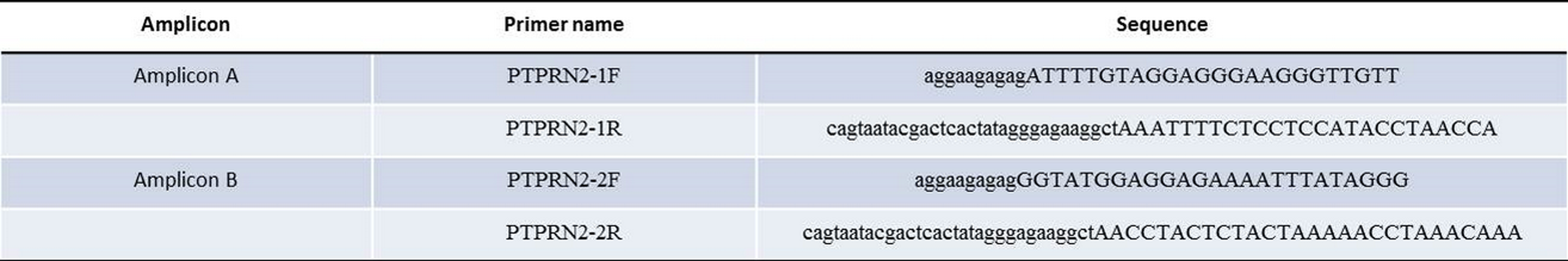

Supplement: S5 Table — (TIF) [file pone.0134119.s005.tif]

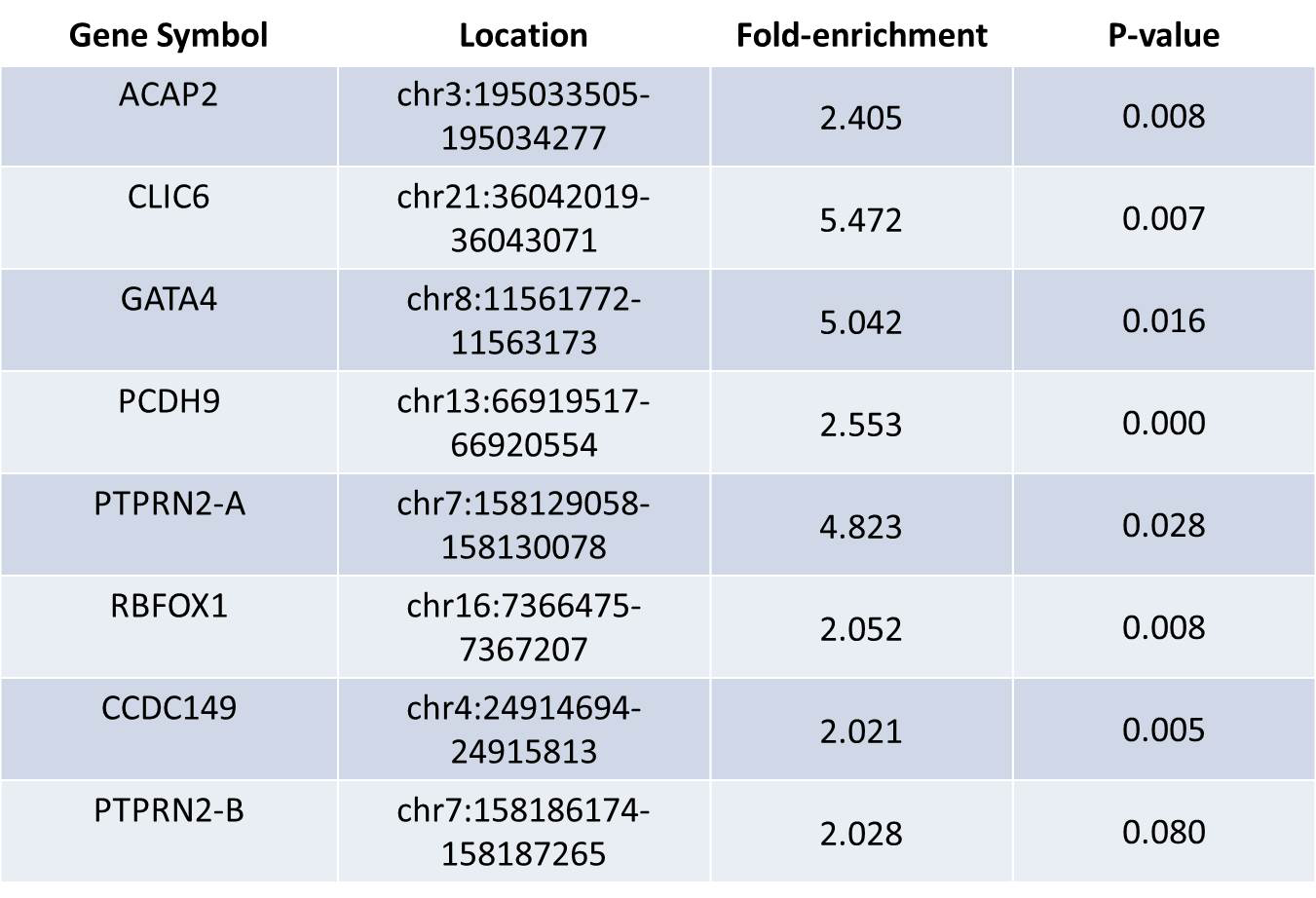

Supplement: S6 Table — (TIF) [file pone.0134119.s006.tif]
